# Nematode Peptides with Host-Directed Anti-inflammatory Activity Rescue *Caenorhabditis elegans* from a *Burkholderia pseudomallei* Infection

**DOI:** 10.3389/fmicb.2016.01436

**Published:** 2016-09-12

**Authors:** Mei-Perng Lim, Mohd Firdaus-Raih, Sheila Nathan

**Affiliations:** ^1^School of Biosciences and Biotechnology, Faculty of Science and Technology, Universiti Kebangsaan MalaysiaBangi, Malaysia; ^2^Malaysia Genome InstituteKajang, Malaysia

**Keywords:** antimicrobial peptides, *B. pseudomallei*, *C. elegans*, immunomodulator

## Abstract

*Burkholderia pseudomallei*, the causative agent of melioidosis, is among a growing number of bacterial pathogens that are increasingly antibiotic resistant. Antimicrobial peptides (AMPs) have been investigated as an alternative approach to treat microbial infections, as generally, there is a lower likelihood that a pathogen will develop resistance to AMPs. In this study, 36 candidate *Caenorhabditis elegans* genes that encode secreted peptides of <150 amino acids and previously shown to be overexpressed during infection by *B. pseudomallei* were identified from the expression profile of infected nematodes. RNA interference (RNAi)-based knockdown of 12/34 peptide-encoding genes resulted in enhanced nematode susceptibility to *B. pseudomallei* without affecting worm fitness. A microdilution test demonstrated that two peptides, NLP-31 and Y43C5A.3, exhibited anti-*B. pseudomallei* activity in a dose dependent manner on different pathogens. Time kill analysis proposed that these peptides were bacteriostatic against *B. pseudomallei* at concentrations up to 8× MIC_90_. The SYTOX green assay demonstrated that NLP-31 and Y43C5A.3 did not disrupt the *B. pseudomallei* membrane. Instead, gel retardation assays revealed that both peptides were able to bind to DNA and interfere with bacterial viability. In parallel, microscopic examination showed induction of cellular filamentation, a hallmark of DNA synthesis inhibition, of NLP-31 and Y43C5A.3 treated cells. In addition, the peptides also regulated the expression of inflammatory cytokines in *B. pseudomallei* infected macrophage cells. Collectively, these findings demonstrate the potential of NLP-31 and Y43C5A.3 as anti-*B. pseudomallei* peptides based on their function as immune modulators.

## Introduction

*Burkholderia pseudomallei* is the causative agent of melioidosis, a fulminant infectious disease prevalent in Northern Australia and Southeast Asia (Wiersinga et al., 2012). The infection triggers various clinical manifestations, ranging from asymptomatic infection to severe septicaemia, in humans and animals. Diagnosis is mainly based on bacterial culture or serological assays whilst treatment is limited to third-generation cephalosporins or carbapenems. No licensed vaccine is currently available for melioidosis although efforts to evaluate the use of live attenuated, inactivated whole cell and recombinant subunits as vaccine candidates are ongoing (Sarkar-Tyson and Titball, 2010). In addition, this pathogen is inherently resistant to a wide range of antimicrobials including ß-lactams, aminoglycosides, and macrolides (Estes et al., 2010), and relapse, recrudescence and high fatality rates are commonly reported even in melioidosis patients administered appropriate and prolonged antibiotics therapy (Stevens and Galyov, 2004). Thus, an alternative approach to address the problem of resistance is the exploitation of antimicrobial peptides (AMPs).

To date, almost 2000 AMPs have been chemically synthesized or identified from various organisms including microbes, insects, invertebrates and mammals (Zhao et al., 2013). They are short, usually cationic peptides of 10–150 amino acids and serve as the first line of defense against pathogenic assault (Jenssen et al., 2006). AMPs have a number of features in common with antibiotics such as the ability to kill bacterial cells and target a wide spectrum of bacteria including clinically relevant antibiotic-resistant pathogens. Whilst antibiotics are susceptible to degradation by bacterial proteases, AMPs are less likely to be successfully targeted by proteases because most of these peptides lack unique epitopes that serve as protease recognition sites (Zasloff, 2002). Many AMPs are known to act directly on the pathogen membrane rendering the development of microbial resistance by mutation less likely (Peschel and Sahl, 2006). Some AMPs are multi-functional with different targets which lowers the possibility of the bacteria acquiring resistance by simply modulating a single target (Marr et al., 2006). Nonetheless, recent findings have demonstrated that members of the genus *Burkholderia*, specifically *B. cepacia*, are highly resistant to AMPs including polymyxin B (Loutet et al., 2011). However, AMPs that are reportedly active against *B. pseudomallei* include the human cathelicidin LL-37 and LL-31, defensin HNP-1, histatin and histatin variants, lactoferrin, bactenecin, cecropin A-magainin (CA-MA), RTA3 and bovine myeloid antimicrobial peptide-18 (BMAP-18; Kanthawong et al., 2012; Madhongsa et al., 2013).

In an earlier study, expression profiling revealed that a group of worm genes that were overexpressed during *B. pseudomallei* infection included a subset of genes with potential AMP function (Lee et al., 2013). The nematode *Caenorhabditis elegans* is thought to produce putative AMPs as part of its inducible defense response toward infection by pathogenic bacteria (Pujol et al., 2008). In this study, potential AMP-encoding genes were knocked down by RNAi and the encoding peptide’s antimicrobial function was assumed if RNAi-treated worms were more susceptible to infection. Peptides that showed potential were synthesized and evaluated using the microdilution test and the underlying mechanism(s) of action of the candidate AMPs as anti-bacterial agents as well as possible immunomodulators was examined in the context of a *B. pseudomallei* infection.

## Materials and Methods

### Bacterial and Nematode Strains

Bacterial strains used were *B. pseudomallei* R15, *Pseudomonas aeruginosa* PA14, *Salmonella typhimurium* SL1344, *Staphylococcus aureus* NCTC8325-4, Methicillin-resistant *S. aureus* (MRSA) ATCC 33591, and *Enterococcus faecalis* V583. Each bacterial culture was aerobically incubated at 37°C unless otherwise stated. The *B. pseudomallei* Human R15 strain used in this study is a biofilm-producing clinical isolate from Malaysia that is pathogenic in the mouse and *C. elegans* infection models (Lee et al., 2007, 2011; Eng and Nathan, 2015). All experiments involving *B. pseudomallei* were performed in a Biosafety Level 2+ laboratory. Standard Operating Procedures for working with pathogens were approved by the Universiti Kebangsaan Malaysia Animal Ethics Committee (UKMAEC) and the Institutional Biosafety Committee. For RNAi feeding, individual *C. elegans* RNAi clones were selected from the Ahringer library (Geneservice, UK; Fraser et al., 2000; Kamath et al., 2003) and the Vidal library (Open Biosystem, USA; Rual et al., 2004). All plasmids were transformed into *Escherichia coli* HT115 (DE3), an RNase III-deficient *E. coli* strain with isopropyl β-D-1-thiogalactopyranoside (IPTG) inducible T7 polymerase activity (Timmons et al., 2001). RNAi sensitive double mutant worms *glp-4(bn2);rrf-3(pk1426)* were obtained from the *Caenorhabditis* Genetics Centre (CGC). Worms were propagated at 16°C on nematode growth medium (NGM) agar pre-seeded with *E. coli* OP50 as the food source.

### Preparation of RNAi-Treated Worms

Gene silencing was performed as previously described (Lee et al., 2013), albeit with minor modifications. Briefly, the axenised eggs of *glp-4(bn2);rrf-3(pk1426)* double mutant worms were spotted onto the bacterial lawn of *E. coli* HT115 expressing dsRNA. The NGM agar plates were supplemented with 100 μg/ml carbenicillin and 1 mM IPTG for RNAi induction. Eggs were allowed to hatch and develop into adult worms over 72 h at 25°C.

### *C. elegans*-*B. pseudomallei* Survival Assay

All assays involving *B. pseudomallei* were standardized according to Ooi et al. (2012). To prepare the bacterial lawn, a single colony of *B. pseudomallei* was inoculated into brain–heart infusion (BHI) medium and grown for 16 h at 37°C at 250 rpm. The overnight culture was spread on NGM agar plates and incubated at 37°C for 24 h. Assay plates were then equilibrated to room temperature for another 24 h. For the survival assay, 120 age-matched RNAi-treated adult worms were transferred onto the bacterial lawn (40 worms/plate) and incubated at 25°C. Worm mortality was scored over time and a worm was considered dead when it was unresponsive to touch with the platinum wire picker. In all experiments, bacteria containing an empty RNAi expression vector (L4440) served as the control. Statistical Kaplan–Meier non-parametric survival analysis was performed using StatView (version 5.0.1; SAS Institute). At least two independent experiments were performed.

### Lifespan Assay

Lifespan assays were carried out as previously described (Lee et al., 2013). Briefly, 120 age-matched adult worms were transferred onto NGM agar plates containing 100 μg/ml kanamycin pre-seeded with *E. coli* OP50, which were exposed overnight to 400 μg/ml kanamycin. Bacteria containing the empty RNAi expression vector (L4440) served as the control. Worm survival was enumerated daily and worms that died due to bursting vulva were censored from further analysis. Statistical Kaplan–Meier non-parametric survival analysis was performed using StatView (version 5.0.1; SAS Institute). At least two independent experiments were performed.

### Peptide Synthesis

All peptides were synthesized using standard 9-fluorenylmethyloxycarbonyl solid phase synthesis (Selleck Chemicals, USA). The peptides were ≥90% pure as confirmed by analytical high-performance liquid chromatography and mass spectrometry. Stock solutions were prepared by dissolving each peptide powder in the solution containing 0.01% (v/v) acetic acid and 0.2% (w/v) bovine serum albumin. Subsequent dilutions were made in test medium and prepared fresh in all experiments.

### Biochemical Properties and Sequence Analysis

The biochemical characteristics for each peptide were predicted using the Antimicrobial Peptide Database^1^ (Wang et al., 2009). Prediction of secondary structure was performed using JPred 3 ^2^ (Cole et al., 2008). Specific regions predicted to form alpha helices were subjected to helical wheel analysis using Heliquest^3^ (Gautier et al., 2008). Atomic structure of peptides was visualized in Jmol v. 13.0 (Herraez, 2006). Spatial orientation of peptides relative to the membrane utilizing the three-dimensional structure program was calculated in the PPM 2.0 server^4^ (Lomize et al., 2012).

### Antimicrobial Susceptibility Test

The broth microdilution test was performed on a panel of bacteria (*B. pseudomallei* Human R15, *P. aeruginosa* PA14, *S. typhimurium* SL1344, *S. aureus* NCTC8325-4, MRSA ATCC 33591 and *E. faecalis* V583) according to the method outlined by the Clinical and Laboratory Standards Institute (CLSI) M07-A9 (Wiegand et al., 2008; CLSI, 2012). Briefly, a single bacterial colony was inoculated into the respective medium supplemented with an appropriate antibiotic (**Table 1**) and grown overnight at 37°C. The overnight culture was centrifuged to remove the supernatant containing selective antibiotics and the pellet was washed with fresh antibiotic-free medium twice. An aliquot of the bacteria suspension was further diluted 1:100 in fresh medium and grown until an OD_600_ = ∼0.5 was reached. The bacterial inoculum size was standardized to approximately 5 × 10^5^ CFU/ml by adjusting the optical density of the bacterial suspension. Serial two-fold dilutions of each peptide were prepared and added into wells of a microtiter plate containing the respective bacterial inoculums. In parallel, two wells with peptide-free medium were used as sterility and growth controls. Plates were incubated at 37°C for 18 h without shaking and read with a microplate spectrophotometer at 630 nm. The MIC endpoint reported is the lowest concentration of peptide able to inhibit the growth of the test organism by 50% (MIC_50_) or 90% (MIC_90_) compared to the growth in control wells. Percentage of growth inhibition was determined using the following formula: [1 - (OD630 of peptide-treated culture/OD630 of growth control)] × 100% (Sherlock et al., 2010). Aliquots from any well with no growth were spotted onto antibiotic-supplemented selective medium agar (**Table 1**) to determine the lowest concentration of the peptide that reduces viability of the initial bacterial inoculum by ≥99.9% [the minimum bactericidal concentration (MBC)]. Two independent replicates of duplicate samples were performed for the experiment.

**Table 1:** Selective medium for bacteria.

Bacterial strain	Selective medium
*Burkholderia pseudomallei* Human R15	BHI agar/broth with gentamycin (4 μg/ml)
*Pseudomonas aeruginosa* PA14	King’s B agar/broth with rifampicin (100 μg/ml)
*Salmonella typhimurium* SL1344	LB agar/broth with streptomycin (50 μg/ml)
*Staphylococcus aureus* NCTC8325-4	TS agar/broth with nalidixic acid (15 μg/ml)
MRSA ATCC 33591	TS agar/broth with nalidixic acid (15 μg/ml)
*Enterococcus faecalis* V583	BHI agar/broth with chloramphenicol (25 μg/ml)

### Time-Kill Assay

Approximately 5 × 10^5^ CFU/ml bacterial cells were exposed to each peptide at final concentrations of 0× MIC_90_, 1× MIC_90_, 2× MIC_90_, 4× MIC_90_, and 8× MIC_90_. Reaction mixtures with and without peptides were incubated at 37°C for 0, 1, 2, 4, 8, and 24 h. At the indicated time points, aliquots were serially diluted in 1× PBS. About 10 μl of each dilution was then spotted on Ashdown agar supplemented with 4 μg/ml gentamycin using the drop plate method with minor modification (Herigstad et al., 2001). Agar plates were incubated at 37°C for 48 h and visually separate colonies were counted. A bactericidal effect was defined as a ≥3 log10 reduction in CFU/ml compared with the initial inoculum. The experiment was performed in duplicate.

### SYTOX Green Assay

The interaction of peptides with *B. pseudomallei* was examined using fluorescence microscopy and an assay based on the uptake of the fluorescent dye SYTOX Green (Invitrogen, USA; Matsumoto et al., 2010) with minor modifications. First, bacterial cells at about 5 × 10^5^ CFU/ml were treated with 8× MIC_90_ peptides for 1 h at 37°C. The cells treated with 0.5% Triton X-100 and 1× PBS were included as the controls. Bacterial suspensions were then stained with 1 μM SYTOX green and incubated for 30 min at room temperature in the dark. An aliquot of the reaction mixtures was spotted onto a glass slide for visualization at 200× magnification under the Leica DM5000B upright microscope equipped with a GFP2 filter cube (bandpass 480/40 nm). Three independent replicates were performed.

### DNA Binding Assay

This test evaluates the presence of peptide-DNA binding by noting the retardation of the rate of migration of DNA bands through agarose gels as previously described (Yan et al., 2013). Briefly, 500 ng of pUC19 plasmid DNA (Invitrogen, USA) was mixed with individual peptides (to final concentrations of between 0.5 and 1024 μM) in 30 μl of 10 mM Tris-HCl, 1 mM EDTA buffer, pH 8.0. Reaction mixtures were incubated at room temperature for 30 min and subsequently electophoresed on 1% agarose gel. The experiment was performed in triplicate.

### Bacterial Filamentation Assay

Bacterial cell morphology was examined to assess if the peptides cause filamentation of *B. pseudomallei* cells, indicative of inhibition of *in vivo* DNA synthesis (Alfred et al., 2013). About 5 × 10^5^ CFU/ml bacterial suspension was exposed to each peptide at 8× MIC_90_. Bacterial cells treated with 1× PBS were used as the negative control. After 1 h incubation at 37°C, an aliquot of the reaction mixture was spotted onto a microscope slide, air dried and stained with crystal violet for 1 min. Excess stain was subsequently rinsed off using distilled water and air-dried. All samples were observed using the Leica DM5000B upright microscope (1000× magnification). Each image was captured using identical settings and the experiment was performed in triplicate.

### Cytokine Measurement

This experiment was conducted according to the protocol described by Thivierge et al. (2013) with modifications. Briefly, ∼2 × 10^5^ cells/well early passage RAW264.7 cells were treated with peptides (0.5 μM each) for 1 h at 37°C. Untreated cells served as the negative control. Cells were washed with 1× PBS and subsequently infected with *B. pseudomallei* at a MOI of 10:1 for an additional 4 h. Cell culture supernatants were harvested and inflammatory cytokines were measured with the multi-analyte ELISArray kit (QIAGEN, Germany). The experiment was performed in duplicate.

### Cytotoxicity Assay

The cytotoxicity of peptides on mammalian cells was examined as described (Thivierge et al., 2013) with minor modifications. In brief, approximately 2 × 10^5^ cells/well early passage murine macrophage RAW264.7 cells were treated with 0, 2.5, 5, 10, 25, 50, 75, 100, 200, and 300 μM peptides for 1 h at 37°C with 5% CO_2_. The culture supernatants were collected and assayed for lactate dehydrogenase (LDH) activity using the CytoTox LDH release kit (Promega, USA). The amount of LDH released was expressed as a percentage relative to the total amount of LDH released from cells treated with lysis buffer. At least two independent replicates were performed for the experiment.

### Statistical Analysis

The survival between RNAi-treated and untreated worms was assessed by the Log-rank (Mantel–Cox) test using StatView (version 5.0.1; SAS Institute). For the other assays, data were expressed as mean ± standard error of the mean (SE) from at least two independent assays. Statistical analysis was performed using the unpaired, two-tailed Student’s *t*-test. The *p*-value of <0.0001 was considered as statistically significant.

## Results

### Identification of Short Peptides Required for Worm Protection against *B. pseudomallei*

**FIGURE 1:**
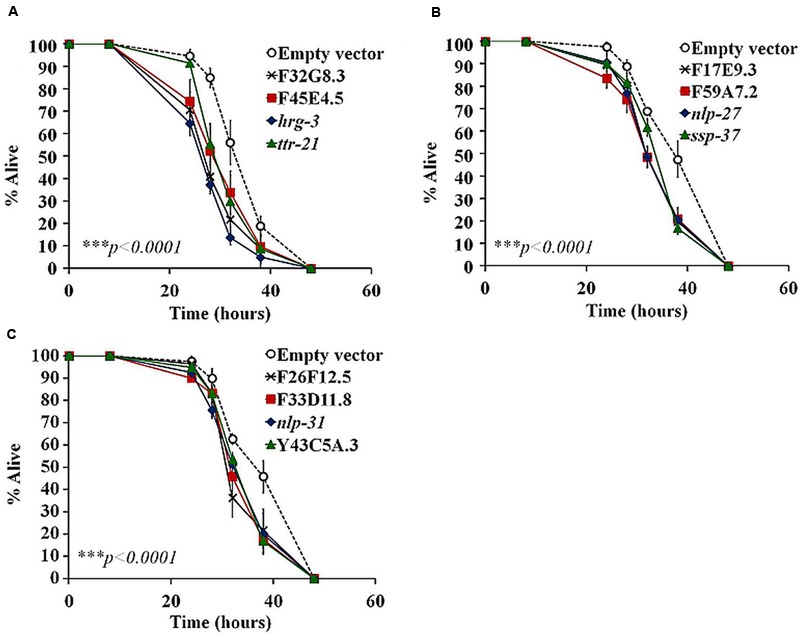
**Functional assessment of putative AMP-encoding gene contribution on worm survival using RNAi feeding.** A time-course of *Burkholderia pseudomallei* infection on RNAi-treated worms was compared to the control. **(A–C)** RNAi-inactivation of candidate genes resulted in an enhanced worm susceptibility to pathogen killing. In a pair-wise comparison to control worms using log-rank tests, the difference is significant (^∗∗∗^*p* < 0.0001). The graph depicts the mean ± SD of three replicates (40 worms/replicate; *n* = 120) from a representative of two independent assays.

In a previous study we analyzed the genome-wide transcriptome of *C. elegans* following *B. pseudomallei* infection (Lee et al., 2013). With the availability of the expression profile, we screened for AMP-like genes that fulfilled the criteria of typical AMPs: (i) genes predicted to encode a peptide of less than 150 amino acids and (ii) carry a signal peptide sequence as determined by signalP 4.0 (Nielsen et al., 1997). The screen identified 36 putative AMP-encoded genes as listed in **Table 2**. To interrogate whether these candidate genes encode peptides that confer protection against the pathogen, 34 of the 36 predicted AMP-encoding genes were individually knocked down in *rrf-3(pk1426);glp-4(bn)* worms by RNAi feeding and mutant worms were challenged with *B. pseudomallei*. The *rrf-3(pk1426)* mutation improves RNAi efficiency (Simmer et al., 2002) whilst the *glp-4(bn)* mutation resulted in the development of sterile adults lacking germ line proliferation, thus eliminating any confounding effects of progeny during infection and analysis (Beanan and Strome, 1992). If these putative AMP-encoded genes are required to protect the worm against *B. pseudomallei* infection, inactivating the genes should present an enhanced susceptibility to pathogen-induced killing (Esp) phenotype. Worms abrogated for the F32G8.3, F45E4.5, *hrg-3, ttr-21*, F17E9.3, F59A7.2, *nlp-27, ssp-37*, F26F12.5, F33D11.8, *nlp-31*, and Y43C5A.3 genes were hypersensitive to infection relative to the vector control (*p* < 0.0001; **Figures 1A–C**; Supplementary Table 2), suggesting that these 12 genes may be involved in the worm antimicrobial response. This set of candidate genes includes *nlp-27* (**Figure 1B**) and *nlp-31* (**Figure 1C**), two representative members of the structurally related neuropeptide-like proteins (NLPs) family of *C. elegans* AMPs. Abrogation of the remaining 22 candidate genes did not lead to a significant difference in nematode survival (*p* > 0.0001; Supplementary Figure 1). These 22 genes include other NLP members, i.e., *nlp-25* (Supplementary Figure 1A), *nlp-29* (Supplementary Figure 1D) and *nlp-30* (Supplementary Figure 1D) whereby RNAi knockdown of the corresponding gene did not alter worm survival during the *B. pseudomallei* infection compared to the control (*p* > 0.0001). This could reflect a redundancy in the function of these NLP members because often, other members of multi-gene families may compensate for loss of the targeted member.

**Table 2:** Putative AMP-encoded genes.

No.	Gene ID	Gene name	Fold change^a^	Size (amino acid)	Signal peptide
1	B0213.2	*nlp-27*	1.35	72	Yes
2	B0213.4	*nlp-29*	3.34	73	Yes
3	B0213.5	*nlp-30*	2.25	69	Yes
4	B0213.6	*nlp-31*	2.72	75	Yes
5	B0334.1	*ttr-18*	1.59	139	Yes
6	C01G10.4		1.24	72	Yes
7	C01G10.5		1.26	75	Yes
8	C23G10.11		0.62	63	Yes
9	C45B2.1		1.45	101	Yes
10	C45B2.2		1.54	99	Yes
11	D2007.1		1.99	111	Yes
12	F09F3.6	*ttr-21*	1.42	145	Yes
13	F10D7.3		2.49	146	Yes
14	F17E9.3		1.48	139	Yes
15	F26F12.5		1.53	118	Yes
16	F32G8.3		1.82	129	Yes
17	F33D11.8		1.12	85	Yes
18	F41E7.4	*fip-5*	1.97	74	Yes
19	F45E4.5		2.14	108	Yes
20	F55B11.4		2.07	145	Yes
21	F58E6.7		2.26	70	Yes
22	F58H10.1		2.38	119	Yes
23	F59A7.2		2.03	116	Yes
24	K04F1.9		4.09	139	Yes
25	K07A1.6^b^		1.77	100	Yes
26	R05A10.4		1.31	112	Yes
27	R09B5.9	*cnc-4*	2.79	66	Yes
28	T08A9.2^b^	*ttr-30*	2.65	143	Yes
29	W02D9.5	*ssp-37*	1.42	112	Yes
30	Y43C5A.3		1.62	108	Yes
31	Y43F8C.1	*nlp-25*	0.81	87	Yes
32	Y46E12A.1	*cnc-6*	5.18	98	Yes
33	Y51A2D.11	*ttr-26*	4.06	144	Yes
34	Y5F2A.2	*ttr-17*	3.26	131	Yes
35	ZK1307.2		1.73	127	Yes
36	ZK970.7		4.31	147	Yes

*^a^Fold change relative to uninfected worms at early time point (4 h post-infection; Lee et al., 2013). ^b^Of the 36 putative AMP-encoded genes, RNAi clones were available for 34 genes and not available for K07A1.6 and T08A9.2.*

To confirm that the Esp phenotype is mediated by infection rather than a consequence of loss of fitness, we silenced these 12 genes individually by RNAi and subsequently assessed worm longevity under normal growth conditions. As *E. coli* OP50 grown on BHI medium is pathogenic to *C. elegans* (Garsin et al., 2001), worms were fed with antibiotic-killed bacteria on NGM agar plates. We confirmed that the observed Esp phenotype was not due to a decrease in worm fitness as the putative AMP-RNAi-treated worms survived as long as the control (*p* > 0.0001; Supplementary Figures 2A–C). Hence, we assume that these 12 genes may potentially encode peptides with antimicrobial property. These genes were then chemically synthesized and used for all subsequent characterization. Peptide sequences and their biochemical characteristics are presented in Supplementary Table 1.

### Antimicrobial Activity of Synthetic Peptides

**FIGURE 2:**
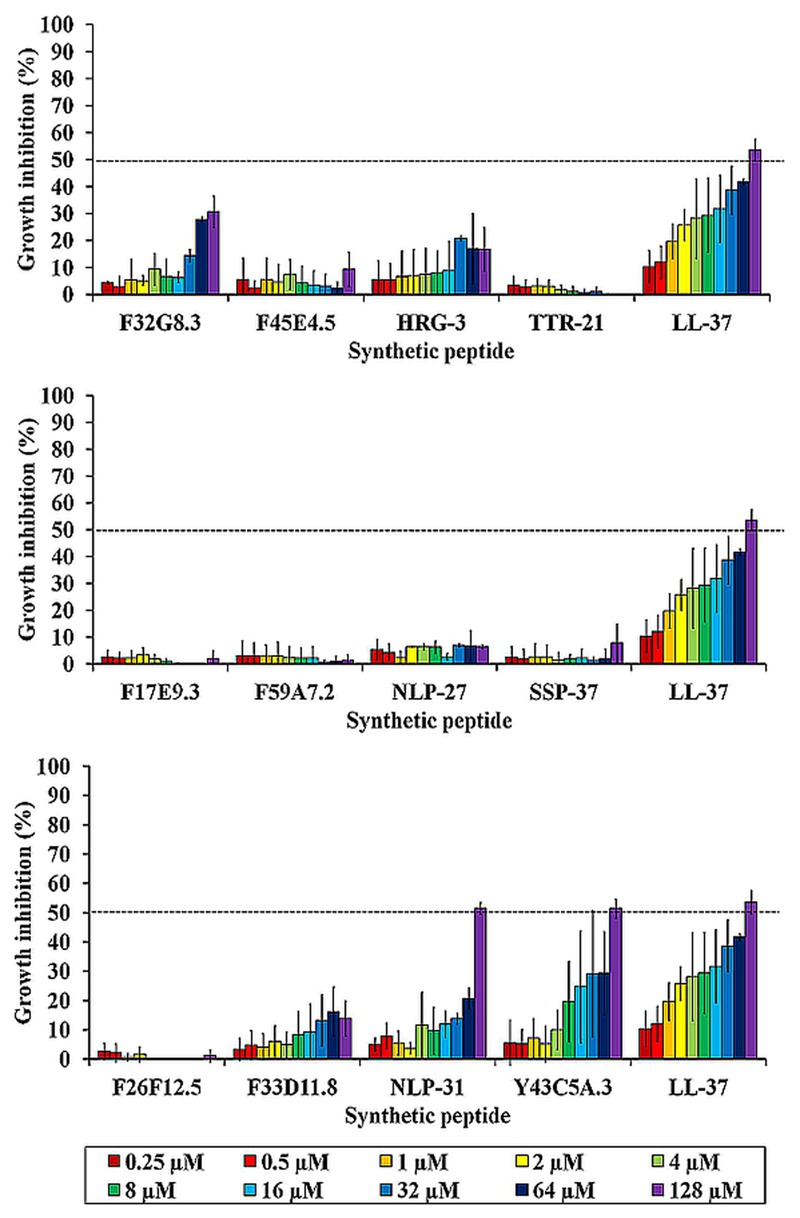
**Anti-*B. pseudomallei* activity of synthetic peptides.** The graph depicts the percentage of bacterial growth inhibition for each peptide at 18 h post-incubation. Results are expressed as mean ± SEM of two individual assays (*n* = 4). Dashed line demarcates the 50% growth inhibition (MIC_50_).

The susceptibility of *B. pseudomallei* to these synthetic peptides (0.25–128 μM) was evaluated through a preliminary microdilution test. To avoid non-specific binding of peptides to the wall of the 96-well plates, polypropylene microtiter plates were used. The control used was the well-described human cathelicidin LL-37 with reported antimicrobial effect on *B. pseudomallei* (Kanthawong et al., 2009). Of the peptides tested, only LL-37, NLP-31, and Y43C5A.3 exhibited anti-*B. pseudomallei* activity in a dose-dependent manner with 50% bacterial growth inhibition at 128 μM (**Figure 2**). No pronounced antimicrobial effect was observed for the other peptides. Next, we asked if these two anti-*B. pseudomallei* peptides exert broad spectrum antimicrobial activity. These antimicrobial effects of NLP-31 and Y43C5A.3 at concentrations ranging from 2 to 1024 μM were further evaluated against a panel of bacteria. Both peptides exhibited antimicrobial activity against all tested microorganisms (**Table 3**), albeit with a more pronounced preference for Gram-negative bacteria.

**Table 3:** Antimicrobial activities of peptides against a broad range of bacteria^∗^.

Bacterium	Gram type	LL-37			NLP-31			Y43C5A.3		
		MIC_50_	MIC_90_	MBC	MIC_50_	MIC_90_	MBC	MIC_50_	MIC_90_	MBC
*Burkholderia pseudomallei* R15	-	128 (576)	256 (1150)	>1024 (>4610)	128 (704)	256 (1410)	>1024 (>5630)	128 (1310)	512 (5220)	>1024 (>10440)
*Pseudomonas aeruginosa* PA14	-	4 (18)	16 (72)	1024 (4610)	64 (352)	256 (1410)	>1024 (>5630)	32 (326.4)	64 (652.8)	>1024 (>10440)
*Salmonella typhimurium* SL1344	-	64 (288)	64 (288)	1024 (4610)	512 (2820)	512 (2820)	>1024 (>5630)	64 (652.8)	256 (2610)	>1024 (>10440)
*Staphylococcus aureus* NCTC8325-4	+	256 (1150)	256 (1150)	>1024 (>4610)	512 (2820)	1024 (5630)	>1024 (>5630)	128 (1310)	512 (5220)	>1024 (>10440)
MRSA ATCC 33591	+	128 (576)	256 (1150)	>1024 (>4610)	512 (2820)	512 (2820)	>1024 (>5630)	1024 (10440)	>1024 (>10440)	>1024 (>10440)
*Enterococcus faecalis* V583	+	128 (576)	128 (576)	>1024 (>4610)	512 (2820)	512 (2820)	>1024 (>5630)	256 (2610)	1024 (10440)	>1024 (>10440)

*MIC_50_: The lowest concentration of peptide (μM) required to inhibit 50% bacterial growth. MIC_90_: The lowest concentration of peptide (μM) required to inhibit 90% bacterial growth. MBC: The lowest concentration of peptide (μM) required to reduce viability of the initial bacterial inoculums by ≥99.9%. Numbers in parentheses represent the corresponding MIC or MBC values in mg/L. ^∗^Data are the average of two replicate experiments performed in duplicates.*

**FIGURE 3:**
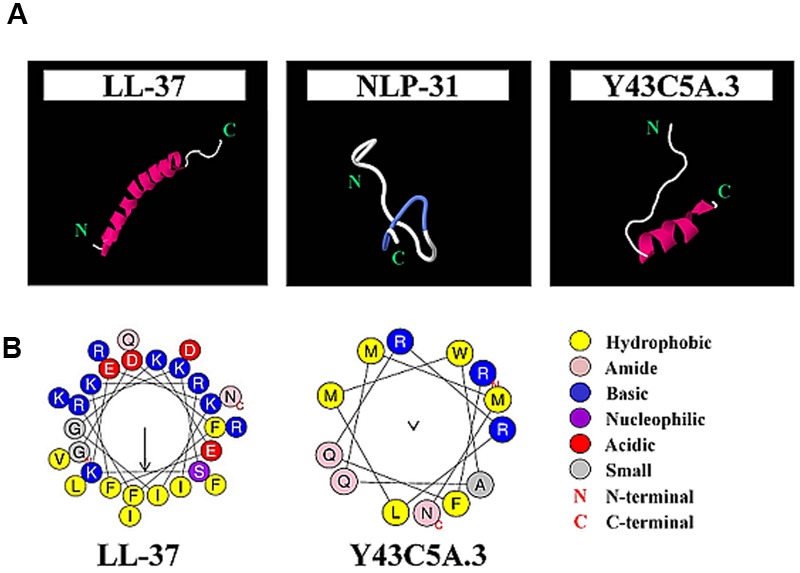
**Secondary structure prediction for LL-37, NLP-31, and Y43C5A.3. (A)** Atomic structure of peptides are shown as cartoon diagrams colored according to secondary structure (violet for α-helix, blue for turns, white for unstructured) and images were produced using Jmol. **(B)** Helical wheel projections for LL-37 (residues 3–30) and Y43C5A.3 (residues 68–80) were generated using Heliquest. Arrows indicate relative hydrophobic moment, a measure of the amphipathicity of peptides in α-helical conformation.

To examine the influence of helicity and amphipathicity on peptide killing activity, peptide structures were predicted followed by subjecting regions containing α-helices to amphipathicity analysis. Both LL-37 and Y43C5A.3 adopted helical structures whilst NLP-31 was in an extended conformation (**Figure 3A**). The amphipathicity of the helical regions for LL-37 and Y43C5A.3 was examined by Heliquest (Gautier et al., 2008). Hydrophobic moment (μH) is used to measure the amphipathicity of a helix and if the value is greater than 0.5, the more likely the α-helical structure will be amphipathic. We found that the helical region of LL-37 (residues 3–30) was clearly amphipathic (μH: 0.629); however, residues 68–80 of Y43C5A.3 were arranged in a non-amphipathic conformation (μH: 0.084; **Figure 3B**). These findings could explain the reduced killing efficacy for both nematode peptides relative to the amphipathic α-helix LL-37 because low helix propensity could lead to a concomitant reduction in antibacterial activity (Nielsen et al., 2007).

### Mode of Action of NLP-31 and Y43C5A.3

**FIGURE 4:**
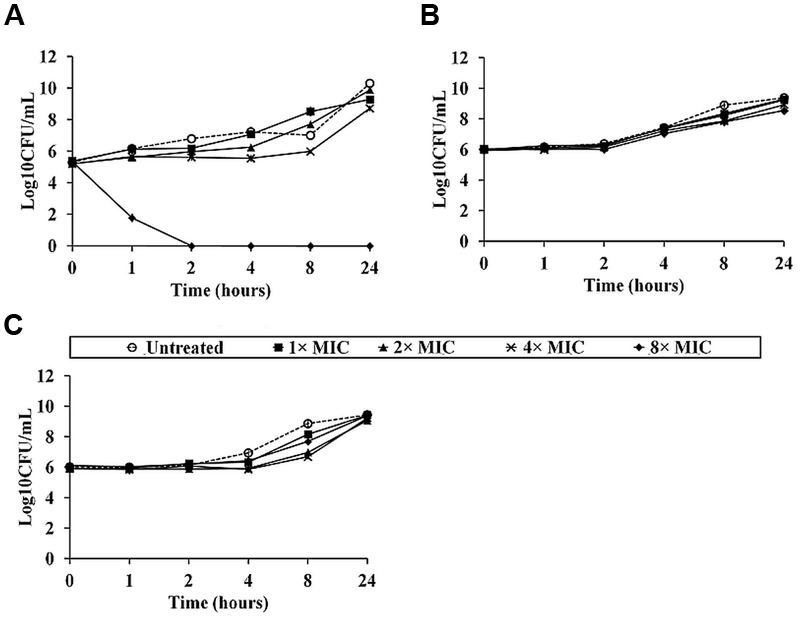
**Killing kinetics of synthetic peptides against *B. pseudomallei*.** Bacterial suspensions were treated with **(A)** LL-37, **(B)** NLP-31 and **(C)** Y43C5A.3 at 0× MIC_90_ (○), 1× MIC_90_ (■), 2× MIC_90_ (▴), 4× MIC_90_ (×) and 8× MIC_90_ (◆) and CFU/ml was enumerated at indicated time points. Untreated bacterial suspension was included as the control. A bactericidal effect was defined as a ≥3 log10 reduction in CFU/ml compared with the initial inoculum. Data are the mean ± SEM of two independent experiments performed in triplicate (*n* = 6).

To examine the *B. pseudomallei* killing kinetics for NLP-31 and Y43C5A.3, bacterial cells were exposed to both peptides at 0× MIC_90_, 1× MIC_90_, 2× MIC_90_, 4× MIC_90_, and 8× MIC_90_ for various time points before determining the number of surviving bacteria at each time point. **Figure 4** depicts the time-kill kinetics of NLP-31 and Y43C5A.3 against *B. pseudomallei*. A reduction in cell viability was observed for cells treated with 8× MIC_90_ of LL-37 within the first 30 min (**Figure 4A**). Complete killing (≥3log10 drop in CFU/ml) was achieved 2 h post-treatment, further supporting the finding that LL-37 possesses bactericidal activity against *B. pseudomallei* (Kanthawong et al., 2012). Conversely, NLP-31 (**Figure 4B**) and Y43C5A.3 (**Figure 4C**) exhibited a bacteriostatic effect on *B. pseudomallei* for the first 4–8 h after incubation.

**FIGURE 5:**
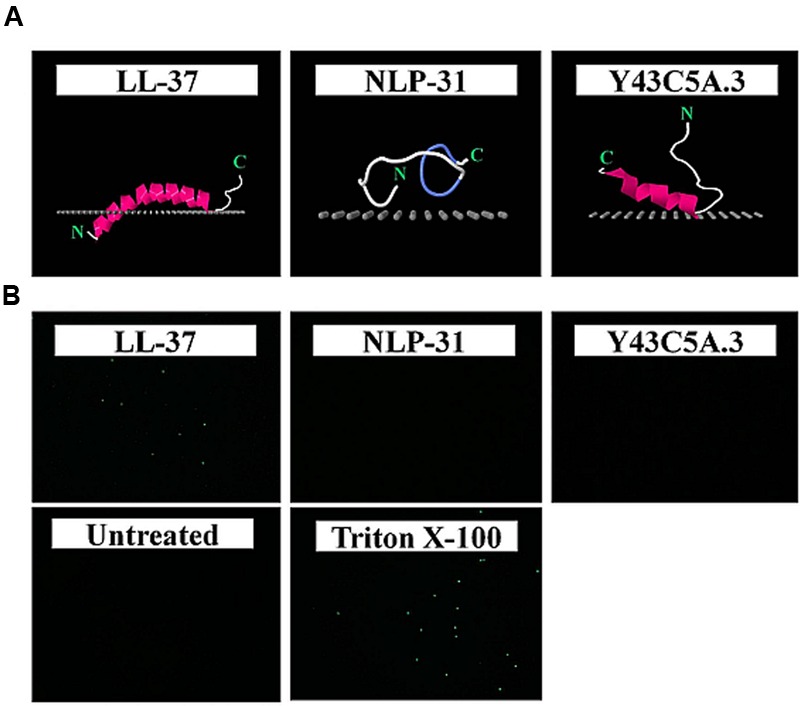
**NLP-31 and Y43C5A.3 do not act against *B. pseudomallei* by disrupting the cell membrane.** Spatial orientation of peptides relative to a simulated membrane were predicted using PPM 2.0 program and validated using the SYTOX green assay. **(A)** Membrane binding models of LL-37, NLP-31, and Y43C5A.3 are shown as cartoon diagrams colored according to secondary structure (violet for α-helix, blue for turns, white for unstructured); the hydrophobic membrane boundary (at the level of the lipid carbonyls) is represented by gray dots. Images were produced using Jmol. **(B)** Representative fluorescence microscopy showing images of *B. pseudomallei* cells treated with 1× PBS (untreated control), 0.5% Triton X-100, 8× MIC_90_ LL-37, 8× MIC_90_ NLP-31, and 8× MIC_90_ Y43C5A.3.

To determine if both peptides interact with the bacterial membrane, an *in silico* approach was used whereby the peptide spatial orientation relative to the inner membrane was calculated using the PPM 2.0 program (Lomize et al., 2012; **Figure 5A**). Modeling of nematode peptide binding on the simulated lipid membrane illustrated that LL-37 inserted deeply into the hydrophobic membrane core (to a depth of 8.6 ± 0.1 Å) whilst NLP-31 and Y43C5A.3 spanned the membrane at a depth of 2.6 ± 1.8 and 2.5 ± 0.9 Å, respectively. The SYTOX green uptake assay was also conducted to study the effects of NLP-31 and Y43C5A.3 on *B. pseudomallei* membrane integrity. This nucleic acid stain is not able to permeate live intact cells, however, if the cell membrane is compromised, the dye binds to DNA as visualized by cytoplasmic fluorescence. No fluorescence was observed in untreated bacteria whilst cells treated with Triton X-100 fluoresced (**Figure 5B**). Similarly, the LL-37 peptide caused an influx of dye into the cytoplasm and SYTOX-stained fluorescent cells were observed corresponding to the earlier report that LL-37 is able to disrupt the membrane of *B. pseudomallei* (Kanthawong et al., 2012). On the other hand, no fluorescence was observed in cells exposed to NLP-31 and Y43C5A.3 proposing that both these peptides adopted a non-membranolytic mode of action. The different mode of action for LL-37 and the nematode peptides is also supported by the accelerated killing kinetics of LL-37 as peptides with better membrane permeability exhibit faster inhibition of bacterial growth (Madhongsa et al., 2013).

**FIGURE 6:**
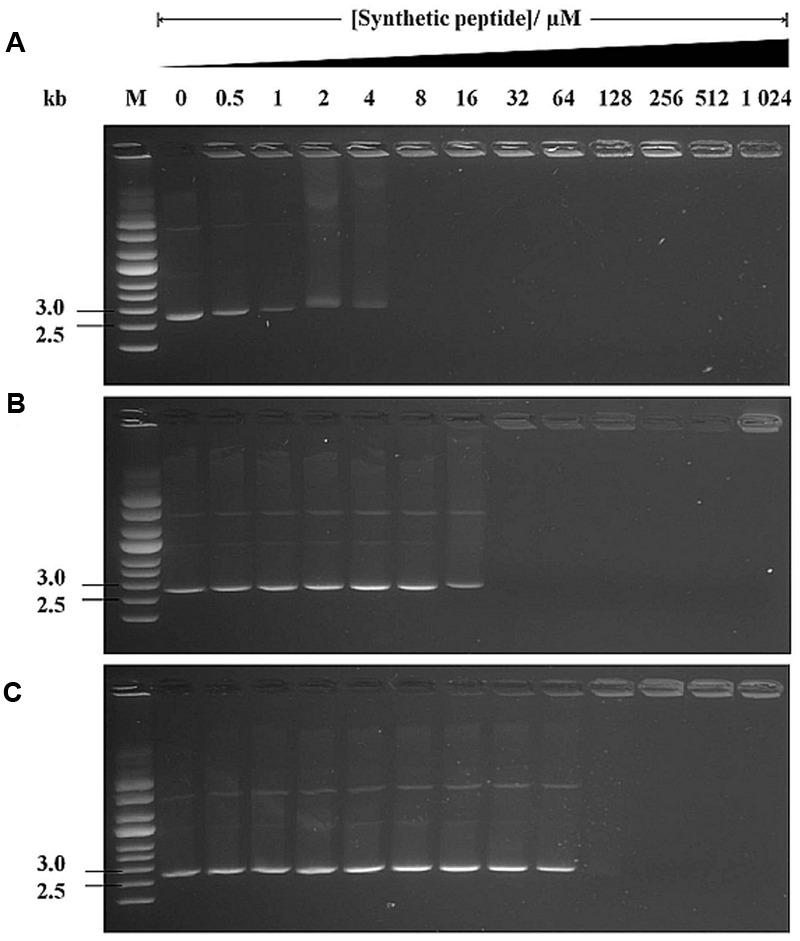
**DNA-binding ability of peptides.** Interaction of **(A)** LL-37, **(B)** NLP-31, and **(C)** Y43C5A.3 with 500 ng of pUC19 plasmid DNA was assessed by measuring the retardation of DNA migration on agarose gels. The number above each lane denotes the concentration of synthetic peptides. Lane M contains the supercoiled DNA ladder.

It is known that some AMPs translocate into the cytoplasm without causing any membrane damage and bind intracellular targets (e.g., DNA), leading to cellular inactivation (Jang et al., 2010). Thus, we investigated the DNA-binding properties of peptides using the gel retardation assay. A fixed amount of supercoiled pUC19 plasmid DNA was mixed with different amounts of peptides and electrophoresed. Plasmid DNA exposed to no peptides or peptides at low concentrations migrated successfully into the gel (**Figures 6A–C**). At higher LL-37 peptide concentrations (8 μM onward), DNA migration was completely retarded (**Figure 6A**). NLP-31 was also able to retard DNA migration from 32 μM onward (**Figure 6B**) whilst as much as 128 μM of Y43C5A.3 was required to inhibit migration of pUC19 (**Figure 6C**). The retardation of DNA migration is most likely attributed to the formation of peptide-DNA complex, indicating that these peptides could interact with DNA with varying affinity and thereafter interfere with the viability of *B. pseudomallei*.

**FIGURE 7:**
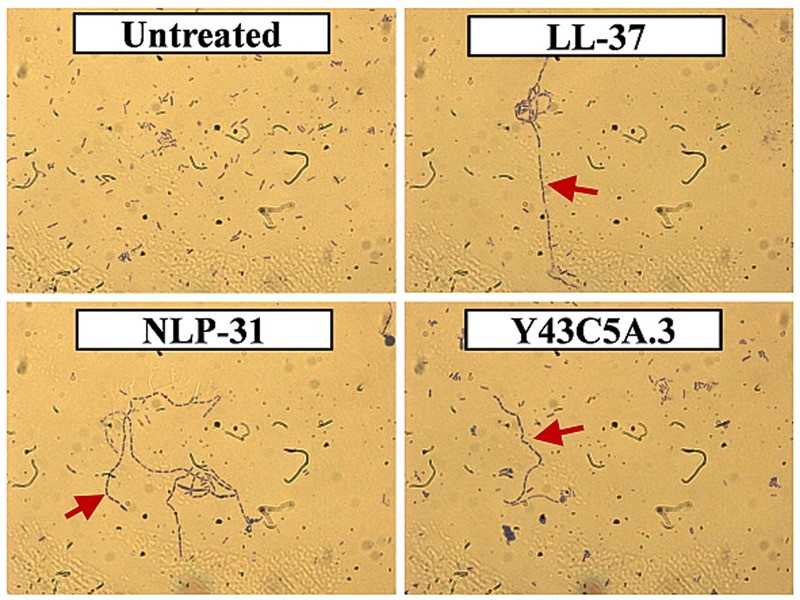
**Morphology of *B. pseudomallei* cells treated with peptides.** Bacterial cells were mixed with individual peptides at 8× MIC_90_ for 1 h at 37°C and observed using light microscopy at 1000× magnification under oil emersion. Representative micrographs of bacteria cells treated with 1× PBS (untreated control), LL-37, NLP-31, and Y43C5A.3 are shown. Red arrow denotes the chain-forming phenotype in *B. pseudomallei* cells.

As AMPs may use multiple mechanisms of action to combat pathogens (Lehrer et al., 1989; de Leeuw et al., 2010), we extended our investigation to determine whether NLP-31 and Y43C5A.3 could inhibit replication *in vivo* by treating bacterial cells with the peptides at 8× MIC_90_ for 1 h and inspecting their morphology by light microscopy. As illustrated in **Figure 7**, the untreated control bacteria appeared as blue rod-shaped individual cells whilst the chain-forming phenotype (denoted by the red arrow) was observed for cells treated with LL-37, NLP-31, and Y43C5A.3. It is known that filamentation can be induced in *E. coli* if antimicrobial agents inhibit bacterial DNA synthesis during cell division causing the rod-shaped cells to continue to grow in size but fail to divide (Lutkenhaus, 1990). Therefore, NLP-31 and Y43C5A.3 bind to DNA and this peptide-DNA complex most likely interrupted the DNA synthesis machinery as reflected by the formation of cellular filaments. Nevertheless, how these peptides are internalized into the bacterial cells still remains unresolved.

### Modulation of Inflammatory Cytokines by NLP-31 and Y43C5A.3

**FIGURE 8:**
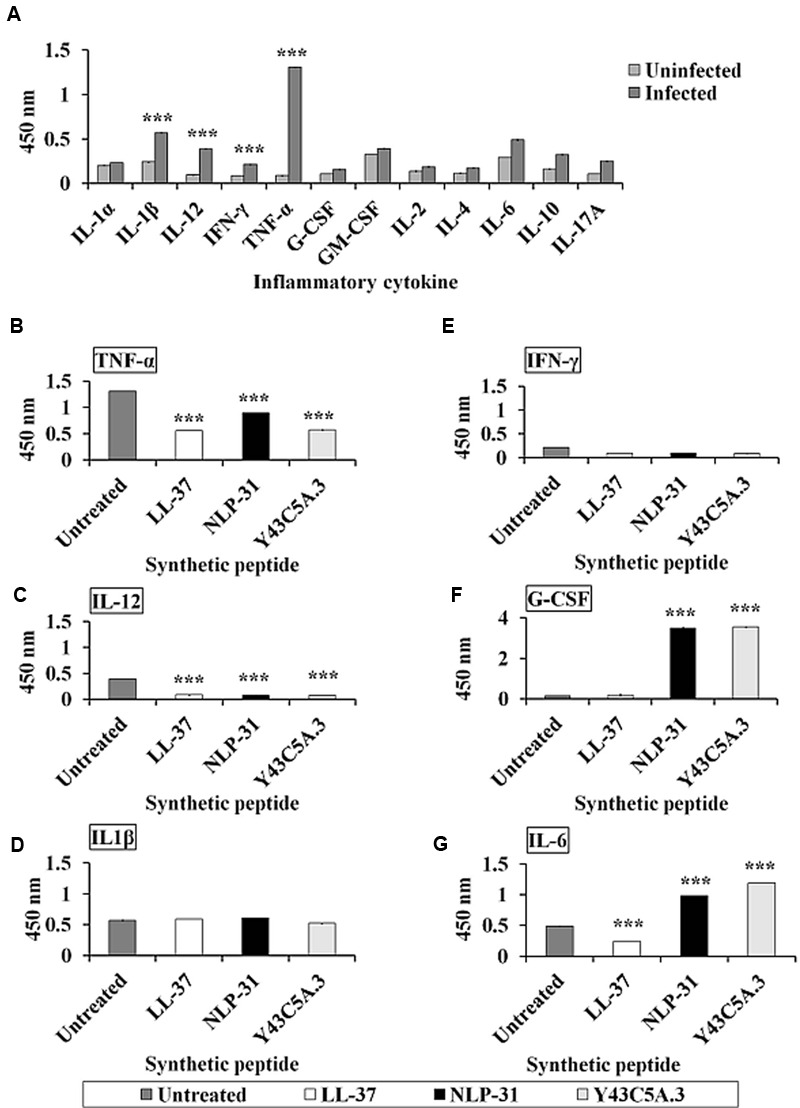
**Peptides modulate the release of pro- and anti-inflammatory cytokines upon *B. pseudomallei* infection. (A)** When challenged with *B. pseudomallei*, elevated levels of several inflammatory cytokines like TNF-α, IL-12, IL-1β, and IFN-γ were detected in infected murine macrophage RAW264.7 cells. However, in the presence of peptides, the secretion of **(B)** TNF-α and **(C)** IL-12 by infected cells was significantly suppressed (*p* < 0.0001) whereas the levels of **(D)** IL-1β and **(E)** IFN-γ remained unchanged. Conversely, unlike LL-37, both nematode peptides significantly induced **(F)** G-CSF and **(G)** IL-6. The data shown are mean ± SEM of two independent experiments (*n* = 4). ^∗∗∗^Significant difference between untreated and treated cells (*p* < 0.0001).

Acute forms of melioidosis generally lead to sepsis and death, both of which are most likely a result of an uncontrolled inflammatory reaction (Gan, 2005). As inflammatory processes are mediated by cytokines, we measured cytokine production in *B. pseudomallei* infected murine macrophage RAW264.7 cells. We noted that the pro-inflammatory cytokines tumor necrosis factor alpha (TNF-α), interleukin (IL)-12, IL-1β and interferon gamma (IFN-γ) were markedly induced upon infection whilst IL-1α, IL-1β, IL-2, IL-4, IL-6, IL-10, IL-17A and granulocyte-macrophage colony-stimulating factor (GM-CSF) were barely detectable (**Figure 8A**). This observation was similar to that previously reported by Chiang et al. (2015).

In this study, murine macrophage RAW264.7 cells were pre-treated with 0.5 μM LL-37, NLP-31 or Y43C5A.3 prior to *B. pseudomallei* infection and the peptide effect on cytokine production was determined. The peptide concentration of 0.5 μM was selected based on the reported physiological concentration for human cathelicidin LL-37 (0.44 μM; Lai and Gallo, 2009). We observed that the levels of TNF-α (**Figure 8B**) and IL-12 (**Figure 8C**) secreted by peptide-treated infected cells were suppressed (*p* < 0.0001) relative to the untreated control cells whereas production of IL-1β (**Figure 8D**) and IFN-γ (**Figure 8E**) remained unchanged. TNF-α and IL-12 are typically pro-inflammatory cytokines and therefore, the observed suppression of these cytokines implies that NLP-31 and Y43C5A.3 may regulate inflammation during sepsis. Moreover, G-CSF (**Figure 8F**) and IL-6 (**Figure 8G**) were induced significantly in NLP-31 and Y43C5A.3 pre-treated cells as compared to the untreated control (*p* < 0.0001). The detection of elevated levels of these cytokines in response to *B. pseudomallei* infection further support the potential application of NLP-31 and Y43C5A.3 as anti-inflammatory molecules.

### NLP-31 and Y43C5A.3 Are Not Cytotoxic to Mammalian Cells

**FIGURE 9:**
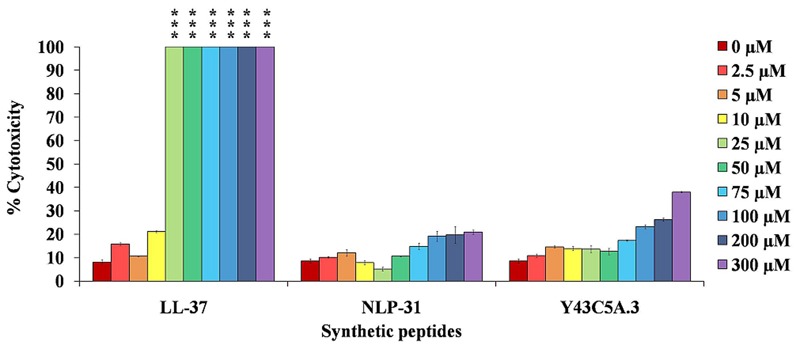
**Nematode peptides are not cytotoxic to mammalian cells.** Murine macrophage RAW264.7 cells were incubated with peptides for 1 h at 37°C. Release of LDH by cells was measured and expressed as the percentage of LDH released after treatment of cells with lysis buffer (regarded as 100%). The bars correspond to the mean ± SEM of two independent experiments performed in triplicate (*n* = 6). ^∗∗∗^A significant difference between untreated and treated cells (*p* < 0.0001).

For AMPs to enter clinical development, it is critical that the peptides are not toxic to the host. We observed that the macrophage cells are vulnerable to ≥25 μM of LL-37 (**Figure 9**), similar to the findings of Thivierge et al. (2013). In comparison, NLP-31 and Y43C5A.3 only exhibited low cytotoxic effects at all the test concentrations up to 300 μM. Moreover, macrophage morphology was normal as observed by light microscopy compared to the shriveled and disrupted LL-37 treated cells (data not shown). LL-37 is more hydrophobic than NLP-31 and Y43C5A.3 (Supplementary Table 1) and it has been established that peptides with greater hydrophobicity are more likely to be toxic to mammalian cells (Bessalle et al., 1993). These nematode peptides with minimal toxicity on eukaryotic cells are ideal candidates for further evaluation as a potential therapeutics.

## Discussion

The growing threat of antibiotic-resistant *B. pseudomallei* and the limited arsenal of antibacterial agents for melioidosis therapy highlight the search for alternative therapeutics. AMPs are good anti-bacterial candidates because the probability that the bacteria will develop resistance is less likely compared to conventional antibiotics (Peschel and Sahl, 2006). A good example of an AMP with limited resistance is nisin, a food preservative which has been used for nearly 60 years (Shin et al., 2015). *B. pseudomallei* is inherently resistant to AMPs such as protamine sulfate, human neutrophil peptide (HNP-1), and polymyxin B (Jones et al., 1996; Burtnick and Woods, 1999). This Gram-negative bacterium employs diverse mechanisms to resist killing by antimicrobial agents, including exclusion from the cell, eﬄux from the cell, enzymatic inactivation, and alteration of target sites (Schweizer, 2012; Bahar and Ren, 2013). Nevertheless, the search for AMPs toward *B. pseudomallei* is being pursued.

Taking advantage of the availability of nematode gene expression data of *B. pseudomallei*-infected worms (Lee et al., 2013), we set out to identify potential nematode AMPs that kill this pathogen. From an initial selection of 36 putative AMP-like genes chosen from the microarray data, a RNA-inhibition screen allowed us to confirm 12 (35%) of these genes as essential to protect the worm against a *B. pseudomallei* infection. Among these, NLP-31 is the only experimentally proven AMP with antibacterial activity against Gram-positive *Micrococcus luteus* and Gram-negative *E. coli* (Couillault et al., 2004). Here, we demonstrated that both NLP-31 and Y43C5A.3 inhibit *B. pseudomallei* growth in a dose-dependent manner. This is the first report on the potential antibacterial property of Y43C5A.3 and more importantly, the ability of both peptides to inhibit *B. pseudomallei*. Previously, Madhongsa et al. (2013) reported that low concentrations (5–20 μM) of bactenecin, CA-MA, RTA3 and BMAP-18 were able to inhibit the growth of *B. pseudomallei* whilst Kanthawong et al. (2012) showed that 100 μM of LL-37 or LL-31 was required for equivalent activity. In our hands, LL-37 inhibited bacterial growth by 90% (MIC_90_) at 256 μM and bactericidal activity was observed within 2 h at 8× MIC_90_. Whilst the previous studies on the effect of LL-37 toward *B. pseudomallei* did not report an experimentally derived MIC value (Kanthawong et al., 2012; Madhongsa et al., 2013), the differences between all three studies could be attributed to differences in the isolates tested and experimental conditions used.

Amongst the microorganisms tested in this study, *P. aeruginosa* PA14 showed relatively high susceptibility to the control peptide LL-37 with an MIC_50_ value of 4 μM (18 mg/L). Neidig et al. (2013) also demonstrated that the MIC value of LL-37 against this bacterial strain was ∼3.56 μM (10 mg/L). In addition, the MIC_50_ value for LL-37 on *S. typhimurium* SL1344 (64 μM, this study) was comparable to that reported by Shprung et al. (2012; >50 μM). AMPs target a wide range of pathogenic bacteria (Gottlieb et al., 2008). Here, we demonstrated that NLP-31 and Y43C5A.3 were capable of exerting broad spectrum antimicrobial activities, with higher selectivity toward Gram-negative bacteria. This is likely due to the difference in outer membrane composition between Gram-positive and Gram-negative bacteria as proposed by Torcato et al. (2013). We also note that the MIC values for *B. pseudomallei* are several magnitudes greater than for *P. aeruginosa* and *S. typhimurium*. This may be attributed to the presence of biofilm or a different type of LPS moiety for *Burkholderia* spp. (Loutet et al., 2011).

Over the past decade, a diverse array of putative AMPs has also been identified based on their induced expression upon infection or sequence similarities (Kato et al., 2002; Pujol et al., 2008), however, the evidence for their biological function is still not fully understood. This study provides information on how the two putative nematode peptides (NLP-31 and Y43C5A.3) exert effects on *B. pseudomallei*. The computational modeling of peptide-membrane interaction predicted that the peptides are able to traverse the cell membrane. Nevertheless, no influx of SYTOX green into the cytoplasm was observed in the presence of peptides, indicating that NLP-31 and Y43C5A.3 are non-membrane acting. Although it is well-documented that most AMPs act primarily by membrane disruption (Matsuzaki et al., 1997), NLP-31 and Y43C5A.3 may be inhibiting *B. pseudomallei* growth through a non-membrane permeabilization mechanism. An alternative to membrane-acting peptides is AMPs that interact with intracellular targets resulting in loss of viability (Nicolas, 2009). For example, the frog AMP buforin II penetrates *E. coli* without destabilizing the cell membrane and inhibits cellular functions by binding to both DNA and RNA (Park et al., 1998). Collectively, we propose that NLP-31 and Y43C5A.3 do not disrupt the bacterial membrane but in turn, interact with cytoplasmic macromolecules to interfere with bacterial viability. This suggestion is supported by the gel retardation assay profile where both peptides were able to bind to DNA and this peptide-DNA complex most likely interrupted the DNA synthesis machinery as reflected by the formation of cellular filaments. The peptides amino acid sequences indicated that both NLP-31 and Y43C5A.3 are glycine/tyrosine-rich peptides. The abundance of glycine and tyrosine may explain the peptides’ DNA binding property. Glycine is a small amino acid with a single hydrogen atom as its side chain could bind to the phosphate moiety on DNA (Schulze-Gahmen et al., 1996). In addition, tyrosine residues can interact with DNA either by hydrophobic interactions via stacking with DNA bases or by hydrogen bonding with the nucleotide through the phenolic OH group (Dimicoli and Helene, 1974).

In addition to direct microbial killing activity, immunomodulatory effects have also been ascribed to AMPs (Afacan et al., 2012). The importance of peptides in defense against infections is partly reflected by their ability to regulate cytokines in macrophage cells. Consistent with this concept, Scott et al. (2002) demonstrated that LL-37 suppressed the LPS-stimulated induction of TNF-α, offering protection in an experimental endotoxemia mouse model. Furthermore, two synthetic AMPs that were developed based on the bovine bactenecin, IDR-1 and IDR-1002, suppressed specific pro-inflammatory cytokines such as TNF-α whilst concomitantly enhancing anti-inflammatory effects (Nijnik et al., 2010). Here, we investigated the potential of NLP-31 and Y43C5A.3 in regulating the inflammatory response triggered by *B. pseudomallei*. Both peptides markedly suppressed the pro-inflammatory cytokines, including TNF-α whilst inducing the anti-inflammatory cytokines. Thus, it is plausible that NLP-31 andY43C5A.3 promote localized immunity to infection whilst preventing the damaging systemic hyperinflammatory response observed in melioidosis patients.

When a melioidosis infection is diagnosed, treatment with appropriate antibiotic therapy is initiated. In general, bacteriostatic agents may be used in the eradication phase of the melioidosis treatment protocol (Inglis, 2010). Although, bactericidal drugs with rapid killing effects offer better clinical outcome, the available evidence suggests that bacteriostatic drugs may be more advantageous for certain infections such as *Streptococcal* toxic shock syndrome (Pankey and Sabath, 2004). We show that NLP-31 and Y43C5A.3 are bacteriostatic toward *B. pseudomallei* only over the first 4–8 h post-incubation.

We suggest that these peptides may be effective if used in combination with antibiotics in the eradication phase of melioidosis treatment at a point when the infected host immune response is strong enough to eliminate the non-replicating bacteria. Recently, Randhawa et al. (2016) have elegantly demonstrated the potential of a cell-penetrating peptide in combination with antibiotic therapy to combat bacterial infection. The cell-penetrating ability and low toxicity of NLP-31 and Y43C5A.3 reported in this study proposes a similar combination of antibiotics and AMPs for melioidosis treatment. This combination therapy may offer an advantage in minimizing antimicrobial resistance, reducing toxic side effects and providing synergistic interaction between the antimicrobial agents (Feng et al., 2015). The peptides efficacy against *B. pseudomallei* can be further evaluated through *in vivo* antibacterial and toxicity effects in infected mice.

## Conclusion

We have identified two *C. elegans* peptides, NLP-31 and Y43C5A.3, that exhibited modest antimicrobial activity against *B. pseudomallei* by interfering with bacterial DNA synthesis and in parallel, showed some promise in modulating host cytokine production to dampen the inflammatory response during infection. The *in vivo* role of these peptides is definitely more complex and may involve immuno-modulation, anti-inflammatory or other host-directed effects rather than direct anti-bacterial effects. Further modifications of these synthetic peptides could possibly lead to enhanced anti-*B. pseudomallei* activities. The non-toxic nature of these peptides toward human macrophage cells urges the evaluation of these AMPs for possible clinical use, particularly against antibiotic-resistant pathogens like *B. pseudomallei*.

## Author Contributions

M-PL and SN conceived and designed the experiments. M-PL performed the experiments. M-PL, SN, and MF-R analyzed the results and wrote the paper.

## Conflict of Interest Statement

The authors declare that the research was conducted in the absence of any commercial or financial relationships that could be construed as a potential conflict of interest.

## Footnotes

1 http://aps.unmc.edu/AP/main.php

2 http://www.compbio.dundee.ac.uk/jpred/index.html

3 http://heliquest.ipmc.cnrs.fr/cgi-bin/ComputParams.py

4 http://opm.phar.umich.edu/server.php
