# Supplementary material for: Nematode Peptides with Host-Directed Anti-inflammatory Activity Rescue Caenorhabditis elegans from a Burkholderia pseudomallei Infection
Source: Front Microbiol. 2016 Sep 12;7:1436. doi: 10.3389/fmicb.2016.01436 (PMC5019075; doi:10.3389/fmicb.2016.01436)
Supplement: Supplementary file 1 [file Table_1.DOCX]

Supplementary Material

Nematode Peptides with Host-directed Anti-inflammatory Activity Rescue *Caenorhabditis elegans* from a *Burkholderia pseudomallei* Infection

Mei-Perng Lim, Mohd Firdaus-Raih, Sheila Nathan*

***Correspondence:** Sheila Nathan: sheila@ukm.edu.my

**Supplementary Table 1. Biochemical properties investigated using Antimicrobial Peptide Database (APD2)**

| **Peptide** | **Net charge^a^** | **Boman index (kcal/mol)^b^** | **Hydrophobic ratio (%)^c^** | **Sequence^d^** |
| --- | --- | --- | --- | --- |
| F17E9.3 | -10 | 2.19 | 33 | ESVLTKFRFNGSFTCSKKVAPFVQKVTVYEHDYLTFDDKLEEVTLFRTDSPDRMSYQFRFEEDGDGKPDDEFYEIYMEIWNTCMIPTENNRVSKYWFKTELPVIDGTLYATENVNLDNI |
| F26F12.5 | +2 | 0.81 | 25 | AVIQSRIQEITNSSPAPSLNKDSSGERVRRQYPYGVGNYGNYGMYNGMYNGMYNGMYGGYGGSPYSYAGYTGYQYPYTNGIFPLQGGYYPGSIVGSGSIWAAGGGLVGNMLSFLIG |
| F32G8.5 | +7 | 1.45 | 30 | GLIQSRVKRQWGGWGGGPGGWGGGPGGWQQPGFQQKNWNKNMNRNHIAAKQDQYNCQTSGINLIGLPISSYNCRNSGMSANVNALQQSHQSQGMMQGPLAGFGGLLNNFMG |
| F33D11.8 | +1 | 3 | 35 | IPLRSLFLRSYDDINQESIARGYFAPQPVDDDNADNRPKRGIDLLKRRVEIIERNRCFFNPITCY |
| F45E4.5 | 0 | 1.07 | 23 | QKEGGMAELKSITGDAPYDGVGNAPAPIPQPMGFWGRPRYSPPGHGFDGPYGGPMMGGGGPFPYSPYNRFTSMSSSPYGLGTWYNNLQ |
| F59A7.2 | -11 | 2.3 | 30 | YPGHHTDHLEGCPLNLTHLHENMTMEHPPRNETRTRRGLDNDIENAGESAVNSVENAGKSAWETAGELGDSGVHTVENAGASVKNGAENLFHEVEAAI |
| HRG-3 | +1 | 1.98 | 31 | RPVMKSGYSKNHHLFRPKNLQTDSEEGFWNNVYFVITASDSFFGG |
| NLP-27 | +4 | 0.38 | 16 | QW**GYGG**MP**YGGYGG**M**GGYG**M**GGYG**M**GY**RRRMW**G**SP**YGGYGGYG**G**YGG**W**G**K |
| NLP-31 | +6 | 1.02 | 5 | QW**GYGG**Y**G**R**GYGGYGGYG**R**GYGGYGGYG**R**GYGGYG**R**G**M**YGGYG**RP**YGG**Y**G**W**G**K |
| SSP-37 | -1 | 0.59 | 46 | AFFRKTLSLTADPPTCTVPATGGFSAHKLVNEFGFVDPFGSKDIVITRTAGAPNWNDKLVIHYAPAPADATDAQAAFAAATLTGQALTIPLTATA |
| TTR-21 | 0 | 1.45 | 33 | VLNVVGSTQTITVTGRLVCQGQPARNVLVKMYEDGTIWDSKLDSTKSANDGTFRVAGTYTKIFTLDPKVNIYHQCNYNGLCSKKLTINIPDYAVASGSGSSTNYDIGTLNLANQFSGETTDCIH |
| Y43C5A.3 | +7 | 1.55 | 14 | Q**YGYY**SP**YYGGGYG**N**YGGYG**N**YY**PS**Y**SS**Y**S**YY**PS**YGY**NN**YGYG**N**YGGYGGY**NN**YGY**NSFGFC**G**IRCRRFQRRLMWAQMMN**YG**M**YG**KK |
| LL-37^e^ | +6 | 2.99 | 35 | LLGDFFRKSKEKIGKEFKRIVQRIKDFLRNLVPRTES |

^a^Charge of peptide at pH 7.0

^b^An estimate of the potential of peptides to bind to other proteins

^c^Ratio of hydrophobic residues/total number of residues (%)

^d^Peptides rich in specific amino acid residues. Y: tyrosine (highlighted in red); G: glycine (highlighted in blue)

^e^Anti-*B. pseudomallei* activity for this human AMP has been described (Kanthawong et al., 2012)

**Supplementary Table 2. The calculated TD_mean_ for RNAi-treated worms infected by *B. pseudomallei***

| **No.** | **Gene ID** | **Gene Name** | **TD_mean_ (hours ± SD)** |
| --- | --- | --- | --- |
| 1 | B0213.2 | *nlp-27* | 36.188 ± 0.746*** |
| 2 | B0213.4 | *nlp-29* | 40.775 ± 0.695 |
| 3 | B0213.5 | *nlp-30* | 38.887 ± 0.748 |
| 4 | B0213.6 | *nlp-31* | 35.823 ± 0.687*** |
| 5 | B0334.1 | *ttr-18* | 39.328 ± 0.687 |
| 6 | C01G10.4 | - | 39.973 ± 0.660 |
| 7 | C01G10.5 | - | 37.692 ± 0.670 |
| 8 | C23G10.11 | - | 37.219 ± 0.573 |
| 9 | C45B2.1 | - | 40.893 ± 0.67 |
| 10 | C45B2.2 | - | 41.462 ± 0.647 |
| 11 | D2007.1 | - | 38.913 ± 0.754 |
| 12 | F09F3.6 | *ttr-21* | 32.574 ± 0.604*** |
| 13 | F10D7.3 | - | 39.952 ± 0.651 |
| 14 | F17E9.3 | - | 35.710 ± 0.711*** |
| 15 | F26F12.5 | - | 35.490 ± 0.698*** |
| 16 | F32G8.3 | - | 30.674 ± 0.653*** |
| 17 | F33D11.8 | - | 36.487 ± 0.767*** |
| 18 | F41E7.4 | *fip-5* | 39.744 ± 0.712 |
| 19 | F45E4.5 | - | 32.807 ± 0.776*** |
| 20 | F55B11.4 | - | 38.871 ± 0.782 |
| 21 | F58E6.7 | *hrg-3* | 29.446 ± 0.564*** |
| 22 | F58H10.1 | - | 37.236 ± 0.684 |
| 23 | F59A7.2 | - | 36.352 ± 0.842*** |
| 24 | K04F1.9 | - | 40.532 ± 0.619 |
| 25 | K07A1.6 | - | ND |
| 26 | R05A10.4 | - | 38.363 ± 0.705 |
| 27 | R09B5.9 | *cnc-4* | 38.363 ± 0.705 |
| 28 | T08A9.2 | *ttr-30* | ND |
| 29 | W02D9.5 | *ssp-37* | 36.754 ± 0.693*** |
| 30 | Y43C5A.3 | - | 36.052 ± 0.639*** |
| 31 | Y43F8C.1 | *nlp-25* | 38.823 ± 0.797 |
| 32 | Y46E12A.1 | *cnc-6* | 40.253 ± 0.797 |
| 33 | Y51A2D.11 | *ttr-26* | 40.104 ± 0.697 |
| 34 | Y5F2A.2 | *ttr-17* | 37.337 ± 0.796 |
| 35 | ZK1307.2 | - | 39.207 ± 0.671 |
| 36 | ZK970.7 | - | 38.541 ± 0.673 |
| 37 | Empty vector (control) | - | 39.593 ± 0.729 |

ND: The worm survival was not determined as the RNAi clones were not available.

****p* < 0.0001 (Log-rank (Mantel-Cox) test)

**Supplementary Figure Legends**

**Supplementary Figure 1. The RNAi-inactivation of candidate genes did not alter worm survival against *B. pseudomallei* infection.** (A-F) Survival curves for RNAi-activated worms with no significant difference in lifespan as compared to the control are presented here (*p* > 0.0001). Graph shows the mean ± SD of three replicates (40 worms/replicate; *n* = 120) from a representative of two independent assays.

**Supplementary Figure 2. RNAi-inactivation does not adversely affect longevity.** (A-C) Survival curves of RNAi-inactivated worms fed with the laboratory food source, *E. coli* OP50. No significant difference in worm survival was observed as compared to the control (*p* > 0.0001). The graph illustrates the mean ± SD of three replicates (40 worms/replicate; *n* = 120) from a representative of two independent assays.
