# Supplementary figures and images for: Nematode Peptides with Host-Directed Anti-inflammatory Activity Rescue Caenorhabditis elegans from a Burkholderia pseudomallei Infection
Source: Front Microbiol. 2016 Sep 12;7:1436. doi: 10.3389/fmicb.2016.01436 (PMC5019075; doi:10.3389/fmicb.2016.01436)

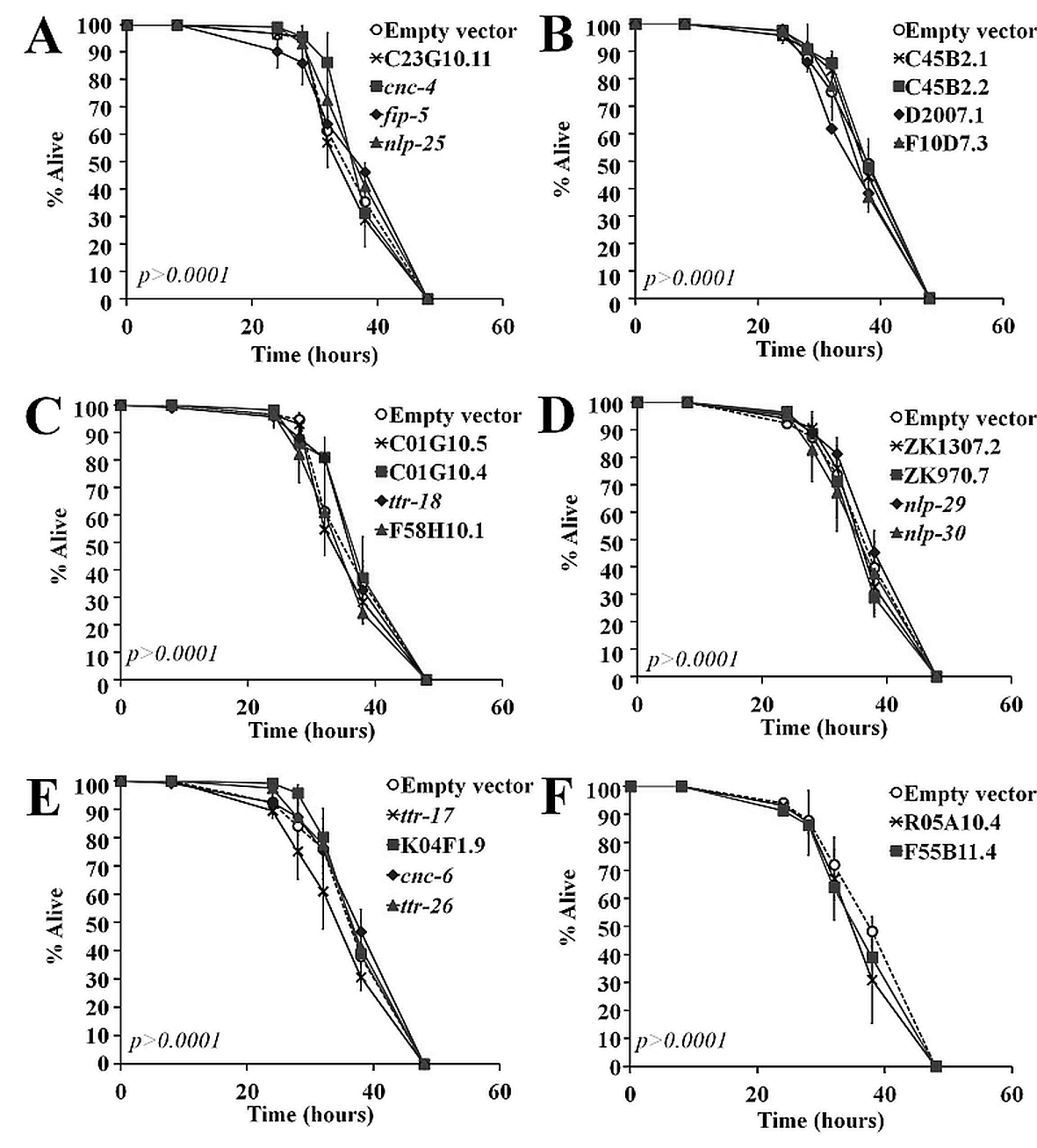

Supplement: Supplementary file 2 [file Image_1.TIF]

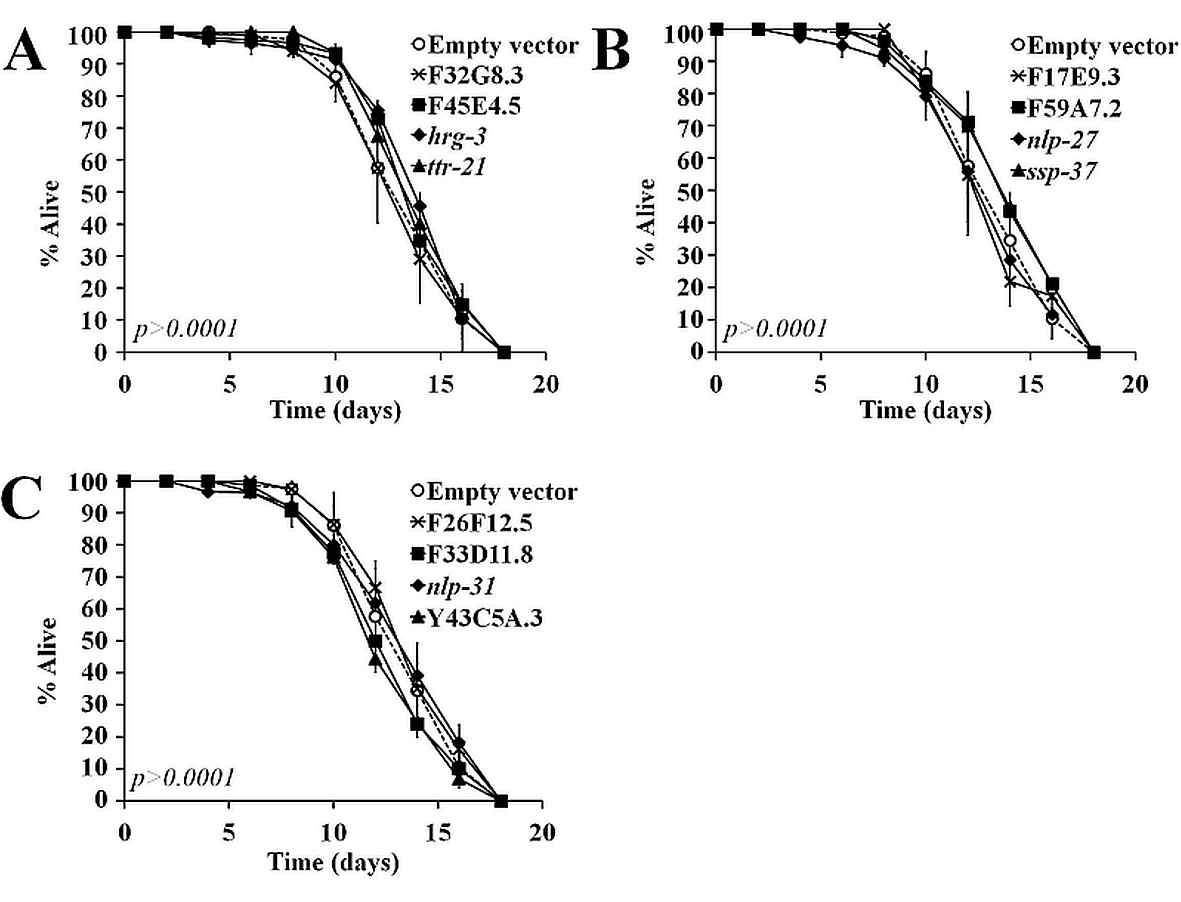

Supplement: Supplementary file 3 [file Image_2.TIF]
